# The analysis of specific antinuclear antibodies concentrations in children with connective tissue diseases

**DOI:** 10.1186/1546-0096-9-S1-P290

**Published:** 2011-09-14

**Authors:** Olga Klochkova, Leila Namazova-Baranova, Ekaterina Alekseeva, Olga Kozhevnikova

**Affiliations:** 1 Scientific Centre of Child Health (RAMS), Moscow, Russian Federation

## Background

Total antinuclear antibodies (ANA) are widely detected in pediatric rheumatology, while the data about specific ANA are few and controversial.

## Aim

To evaluate the levels of ds-DNA, Sm, anti-Ro, anti-Jo1, Scl-70 antibodies in serum of children with connective tissue diseases (CTDs). To look for correlations between clinical features of the CTDs, age and gender of children and specific ANA concentrations.

## Methods

We observed 187 children from 2 to 18 years (median 13 years, 25‰ – 9 years, 75‰ – 15 years); 70 (37,4%) boys, 117 (62,6%) – girls with systemic lupus erythematosis (SLE), juvenile rheumatoid arthritis (JRA), spondyloarthritis (SA), scleroderma (systemic/localized), dermatomyosites and mixed CTD (MCTD). All patients undergo common rheumatologic examination. Specific ANA were measured with Elia method on ImmunoCAP 250 (Phadia).

## Results

Only girls with CTDs had positive specific ANA. There were no significant differences between the ages of children with positive and negative results and with different positive antibodies. Duration of the disease and concentration of antibodies didn’t correlate. Children with positive results had severer forms of CTDs, but there were also 4 patients with active SLE and negative results of measured antibodies. Anti-Ro were detected in the «above» concentrations in 7 cases (all with SLE) and anti-Jo-1 - in all positive cases. 7 children with SLE had combination of positive ds-DNA and Sm or ds-DNA and anti-Ro. (Table 1)

**Table 1:** 

ANA	Number of positive results	Negative results (median concentration; 25‰; 75‰)
dsDNA	19 (10,1%) (17 SLE, 1 JRA, 1 scleroderma)	1 (0,4;2) (IU/ml)

Sm	4 (2%) (4 SLE)	0,1 (0;0,3) (U/ml)

anti-Ro	13 (7%) (10 SLE, 2 MCTD, 1 systemic scleroderma)	0,1 (0;0,3) (U/ml)

anti-Jo1	2 (1%) (2 MCTD)	0 (0; 0,1) (U/ml)

Scl-70	0	0 (0;0,1) (U/ml)

## Conclusion

Ds-DNA, Sm, anti-Ro, anti-Jo1 were found predominantly in girls with SLE. The levels of antibodies didn’t correlate with age and duration of the disease. Evaluation of Scl-70 was not indicative for systemic or localized scleroderma. Further observations are necessary to evaluate the prognostic value of measured specific ANA in these children.

